# Nano-Iron (III) Oxide-Doped Poly (Itaconic Acid-Co-Acrylamide)/Sodium Alginate Hydrogel for Saline–Alkali Soil Amelioration and Wheat Growth

**DOI:** 10.3390/gels12060558

**Published:** 2026-06-22

**Authors:** Zhaomin Sang, Wenhui Zhang, Qinghua Jia, Jianping Zhang, Huiping Ding, Yaling Lu, Ming Ou

**Affiliations:** 1 Engineering Laboratory of Chemical Resources Utilization in South Xinjiang of Xinjiang Production and Construction Corps, Tarim University, Alar 843300, China; szm00425@163.com (Z.S.); 18354785662@163.com (W.Z.); qing_hua13@163.com (Q.J.); dinghuiping131@163.com (H.D.); 2 Analysis and Testing Center, Tarim University, Alar 843300, China; 3 Xinjiang Production & Construction Corps Key Laboratory of Protection and Utilization of Biological Resources in Tarim Basin, Tarim University, Alar 843300, China; jpz2008@126.com

**Keywords:** itaconic acid, soil conditioning properties, pH regulation, wheat cultivation

## Abstract

Soil salinization poses a significant global challenge to agriculture and the environment, leading to decreased soil fertility and hindered crop growth. Therefore, the development of effective and environmentally friendly soil improvement strategies is crucial for sustainable agriculture. In this study, a range of eco-friendly, versatile, and highly absorbent hydrogels for soil enhancement were created using itaconic acid (IA) as a hydrophilic monomer. Furthermore, their effectiveness and application in agriculture were thoroughly evaluated. The nano-iron-loaded IA-based hydrogels (nano-iron (III) oxide (nano-Fe_2_O_3_)/Poly itaconic acid (PIA)-Acrylamide (AM)/Sodium alginate (SA)) hydrogels demonstrated exceptional water absorption and retention capabilities. They exhibited remarkable soil conditioning properties by leveraging carboxyl groups for electrostatic adsorption of saline ions and the porous structure created by the crosslinked network. These features not only significantly facilitated gradual regulation of pH levels and salinity but also effectively enhanced organic matter in saline–alkali soil. Meanwhile, nano-Fe_2_O_3_ simultaneously served to stabilize the hydrogel structure and enhance crop nutrient absorption. Wheat cultivation trials demonstrated that the hydrogels notably enhanced the growth of 7-day-old wheat seedlings. The degradation rates of the hydrogels can be adjusted by varying the IA amount, allowing for the continuous release of small organic molecules to enhance soil quality, aligning with various crop growth cycles. Overall, these hydrogels function as environmentally friendly and versatile soil conditioners, offering significant potential for enhancing agricultural soil quality and expanding into related fields.

## 1. Introduction

The continuous increase in saline–alkali land area has been estimated at approximately 900 million hectares, accounting for one-tenth of the world’s arable land [1]. This expansion can cause extensive and far-reaching damage to agriculture and the ecological environment. Issues such as the deterioration of soil water-holding capacity and the exacerbated leaching of chemical fertilizer nutrients have become increasingly evident [2], culminating in a significant decline in arable land productivity, with crop yield penalties ranging from 30% to 50% [3].

These interrelated environmental and agricultural challenges have also triggered eutrophication of surrounding water bodies [4], perturbed the equilibrium of aquatic ecosystems, and even jeopardized the safety of agricultural products and human health via the soil–crop migration pathway [5]. Currently, saline–alkali soils in arid and semi-arid regions, owing to their high pH and saline characteristics, have drastically impeded the uptake of water and nutrients by crop roots. Specifically, this constraint manifested as osmotic stress induced by hypersaline environments, and suppressed nutrient ion uptake attributable to elevated pH values [6]. However, conventional amelioration strategies such as the application of gypsum and the supplementation of humic acid generally have suffered from drawbacks such as delayed efficacy and the necessity for extensive deployment [7], posing challenges to achieving long-term soil amelioration [8]. Similar limitations were also observed in semi-arid saline soil improvement, where conventional conditioners failed to balance water retention and ion regulation efficiency [9]. This has emerged as one of the pivotal challenges currently hampering the sustainable development of global agriculture [10].

To date, bio-based hydrogels have achieved significant advancements in agricultural soil improvement on account of their combined environmental friendliness and functionality [11]. Research has indicated that hydrogels synthesized by copolymerizing cellulose extracted from wheat straw with acrylic acid can enhance soil water-holding capacity, facilitate controlled water release under drought conditions, and effectively promote maize growth [12]. Following alkaline treatment, rice husk cellulose was graft-copolymerized with polyacrylic acid, which maintained stable water retention capacity even in hard water environments and notably improved the germination rate of fenugreek seeds [13]. Natural polysaccharide composite hydrogels exhibit diverse functions. The ionically crosslinked hydrogel composed of κ-carrageenan, sodium alginate (SA), and carboxymethyl cellulose exhibited a water absorption rate of 2000%, which can amplify soil water-holding capacity by 17.4% and enable slow-release of fertilizers [14]. Additionally, the composite hydrogel of konjac glucomannan and pectin can recover phosphorus from wastewater and promote seed germination [15], thereby establishing an agricultural “purification-reuse” cycle [16]. However, these natural polysaccharides have weak water retention capacity in high-salt environments, which limits their adaptability. Furthermore, the acquisition cost of natural polysaccharides with favorable swelling performance remains relatively high [17], posing challenges for mass production.

IA serves as a vital biomass platform compound extensively utilized in biodegradable polymers due to its non-toxic [18], cost-effective, biodegradable, and renewable characteristics [19]. IA polymers find applications as water retainers, superabsorbent polymers, and slow-release fertilizer coatings [20]. Nevertheless, despite its extensive industrial applications, research on IA and its composites for improving saline–alkali soils remain limited. The acidity, water, and nutrient retention abilities, easy polymerization, and affordability of IA-based polymer hydrogels suggest a significant role in ameliorating saline–alkali land. Additionally, nano-Fe_2_O_3_ has attracted attention for its distinctive properties, it enhances crosslinking to strengthen the hydrogel network, improves thermal stability, and synergistically boosts the hydrogel’s salt regulation performance through electrostatic interactions and coordination with saline ions. Furthermore, its micronutrient characteristics promote soil nutrient conversion and support crop growth [21,22].

Based on the above considerations, novel saline–alkali soil conditioners, nano-Fe_2_O_3_ doped poly (IA-co-acrylamide (AM))/SA hydrogels, were formulated and characterized. Their applications both in wheat seedlings and saline–alkali soils were considered. The soil amendment materials of IA-based hydrogels had the advantages of environmentally friendly, simple preparation processes, and multi-functional synergy of water retention and salt control. They can achieve a balance between remediation effectiveness and economic feasibility, thereby providing a promising strategy for the global remediation of saline–alkali and desertified soils.

## 2. Results and Discussion

### 2.1. Morphology and Structure Characterization of Hydrogels

#### 2.1.1. SEM and EDS Analysis

**Figure 1:**
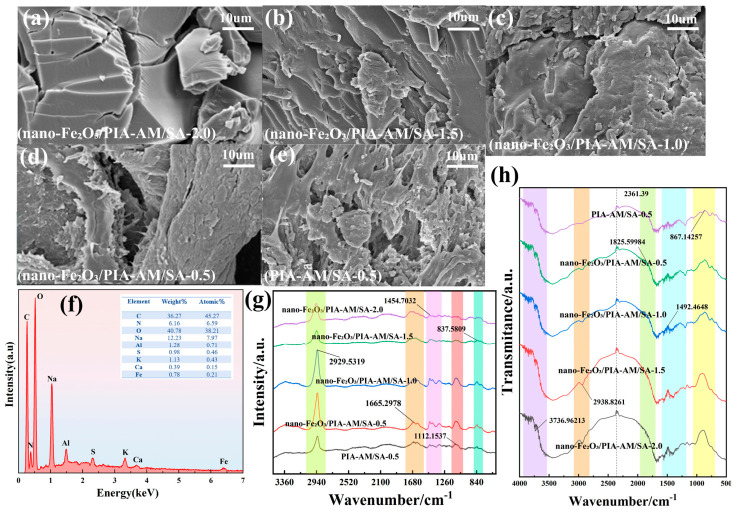
SEM micrographs (**a**–**e**), EDS image of nano-Fe_2_O_3_/PIA-AM/SA-1.5 (**f**), Raman (**g**), and FTIR spectrum (**h**).

SEM visually displays the surface morphology characteristics of the synthesized hydrogels. Figure 1a–e present the SEM of PIA-AM/SA-0.5 and nano-Fe_2_O_3_/PIA-AM/SA series samples with different IA contents, respectively, all exhibiting inherent porosity and large specific surface area. Compared with nano-Fe_2_O_3_/PIA-AM/SA-0.5 and PIA-AM/SA-0.5, nano-Fe_2_O_3_/PIA-AM/SA-2.0 featured a distinct morphological difference. Nano-Fe_2_O_3_/PIA-AM/SA-0.5 and PIA-AM/SA-0.5 exhibited a loose porous structure with thin pore walls, evenly distributed and mutually connected interstices, and a moderately rough surface. The incorporation of iron (III) did not alter the pore architecture but made the surface appear more elastic and resilient. Notably, all nano-Fe_2_O_3_/PIA-AM/SA samples maintained the fundamental gel network structure after adjusting IA dosage, indicating that IA content mainly modulated pore size and surface roughness rather than destroying the gel framework. At the microscale, this porous and rough surface feature is critical for water absorption performance, as the expanded pore channels facilitate efficient diffusion of water molecules into the hydrogel network. Meanwhile, the surface morphology of hydrogels significantly affected their specific surface area and water contact efficiency. In contrast, nano-Fe_2_O_3_/PIA-AM/SA-0.5 and PIA-AM/SA-0.5 demonstrated superior water absorption capacity, while nano-Fe_2_O_3_/PIA-AM/SA-2.0 exhibited relatively poor performance. This difference can be ascribed to the loose porous nanostructure and rough surface developed under low IA dosage. Conversely, elevated IA content led to the formation of a dense and smooth surface on nano-Fe_2_O_3_/PIA-AM/SA-2.0, limiting water diffusion and retention.

Figure 1f presents the EDS analysis of the synthesized nano-Fe_2_O_3_/PIA-AM/SA, which revealed characteristic peaks of C, N, O, Na, Al, S, K, Ca, and Fe. This confirmed that the preparation process preserved the core organic polymer network and nano-Fe_2_O_3_, while maintaining the stable form of key raw material-derived elements. Notably, the O peak (40.78 wt%, 38.21 At%) corresponded to oxygen-containing functional groups, for instance, carboxyl groups in IA/SA, and amides in AM, which were critical for water absorption. The N peak (6.99 At%) aligned with the theoretical dosage of AM, thereby verifying its successful crosslinking into the hydrogel framework. The Fe peak (0.21 At%) confirmed stable loading of nano-Fe_2_O_3_, as indicated by the absence of peak shifts, which suggested an unchanged chemical environment. Additionally, the intense C peak (36.27 wt%) corroborated the formation of a dense organic polymer network. Trace elements, including Na and Al, originated from raw material impurities without interfering with the main composition. The absence of shifts in the core element peaks confirmed the intact chemical environment of both organic and inorganic components. This elemental distribution aligned with reported organic–inorganic composite hydrogel systems, where the integration of oxygen-rich organic groups and inorganic Fe components synergistically underpins the material’s superior water absorption performance, as directly corroborated by this EDS analysis.

#### 2.1.2. FTIR and Raman Spectroscopic Analysis

Raman spectra of the hydrogels were present in Figure 1g. The Raman peak at 2929 cm^−1^ was assigned to the stretching vibration of saturated C-H bonds, originating from the -CH_2_- moieties of AM and the alkyl chains of IA. The characteristic Raman peak at 1665 cm^−1^ corresponded to the stretching vibration of C=C double bonds, derived from residual carbon–carbon double bonds in IA molecules (or unpolymerized monomers). Notably, the nano-Fe_2_O_3_/PIA-AM/SA-2.0 sample unveiled a significantly enhanced intensity of this peak, a phenomenon that exhibited a direct positive correlation with the feeding amount of IA monomer. The Raman peak at 1455 cm^−1^ was attributed to the bending vibration of C-H bonds (methyl and methylene groups), acting as an auxiliary vibrational signal for the saturated hydrocarbon structure. This further confirmed the existence of the hydrocarbon backbone and enhanced the structural stability of the hydrogel through intermolecular van der Waals forces. The Raman shift at 1112 cm^−1^ was assigned to the stretching vibration of C-O-C ether linkages or the skeletal vibration of C-C bonds, originating from the ether linkages of SA and the carbon chains formed by the polymerization of AM and IA. The characteristic Raman peak centered at 838 cm^−1^ corresponded to the vibrational signal of Fe-O bonds, which was indicative of nanosized Fe_2_O_3_ loaded in the hydrogel, confirming the successful combination of Fe_2_O_3_ with the organic matrix. In contrast, PIA-AM/SA-0.5 lacked this characteristic peak, further verifying that this peak serves as a specific loading signature for nanosized Fe_2_O_3_. Collectively, these characteristic Raman peaks reflected the composite structure of the hydrogel, where each structural unit acts synergistically to guarantee fundamental performance and enable functional tunability.

Figure 1h shows the FTIR spectra of series of hydrogels, and the structural information revealed by their characteristic peaks was consistent with the results of Raman spectroscopy. The minor peak at 3737 cm^−1^ corresponded to the stretching vibration of unbound hydroxyl (-OH) groups, present in all samples, originating from the carboxyl hydroxyl groups of IA and the hydroxyl groups of SA. These groups could enhance the hydrophilicity and swelling property of the hydrogel, provide binding sites for soil salt ion adsorption. The peak at 2939 cm^−1^ corresponds to the stretching vibration of saturated C-H bonds. The peak intensity of PIA-AM/SA-0.5 was significantly weaker than that of the nano-Fe_2_O_3_/PIA-AM/SA series, indicating that the hydrogels containing nano-Fe_2_O_3_ possessed a greater abundance of saturated hydrocarbon structures derived from the alkyl chains of AM and IA. This backbone provided mechanical strength for the hydrogel, supported the polymerization and crosslinking of organic components, and thus ensured structural stability. The peak at 1492 cm^−1^ was associated with the methyl structures in AM and IA, and the peak at 1403 cm^−1^ corresponds to the bending vibration of carboxyl groups. The both peaks jointly consolidated the organic backbone and affected the mechanical properties and swelling behavior of the hydrogel through intermolecular interactions. The peak at 867 cm^−1^ corresponds to the vibration of Fe-O bonds, which was only detected in the nano-Fe_2_O_3_/PIA-AM/SA series, confirming the successful loading of nano-Fe_2_O_3_. Complementary FTIR and Raman characterization clearly verified the composite structure of the hydrogel and provided a structural basis for its functional regulation.

#### 2.1.3. XPS Analysis

**Figure 2:**
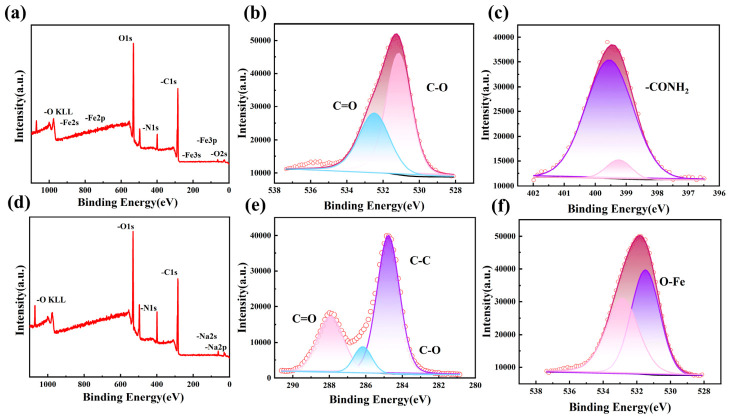
(**a**,**d**) are full-scan survey spectra of nano-Fe_2_O_3_/PIA-AM/SA-1.5 and PIA-AM/SA-0.5, respectively; (**b**,**c**,**e**,**f**) are high-resolution O 1s, Fe 2p, N 1s, C 1s spectra.

Figure 2 displays the spectra of various series hydrogels obtained from X-ray photoelectron spectroscopy. The full-scan survey spectrum of nano-Fe_2_O_3_/PIA-AM/SA-1.5 (Figure 2a) revealed distinct characteristic peaks of O 1s, C 1s, N 1s, Fe 2p/3p, and O KLL, the oxygen Auger electron peak, confirming the successful loading of nano-Fe_2_O_3_. In contrast, the survey spectrum of PIA-AM/SA-0.5 (Figure 2d) exhibited signals of O 1s, C 1s, N 1s, Na 2s/2p, and O KLL without Fe-related peaks, which verified that Fe originated from the introduced nano-Fe_2_O_3_. Detailed structural information was provided by high-resolution spectra. The C 1s spectrum (Figure 2e) is deconvoluted into peaks at 284.8 eV for C-C, 286 eV for C-O, and 288 eV for C=O, corresponding to the carbon chain, hydroxyl/carboxyl, and carbonyl structures, respectively. The N 1s spectrum (Figure 2c) reveals a peak at 399–400 eV assigned to the -CONH_2_ of AM. The O 1s spectrum of PIA-AM/SA (Figure 2b) shows peaks corresponding to C-O and C=O bonds derived from the carboxyl of IA and the hydroxyl of SA. Additionally, the O 1s spectrum of nano-Fe_2_O_3_/PIA-AM/SA (Figure 2f) exhibits an extra peak at 530 eV attributed to O-Fe bonds, indicating the coordination interaction between the hydrogel matrix and nano-Fe_2_O_3_.

#### 2.1.4. TGA, DGT, XRD and Contact Angle Analysis

**Figure 3:**
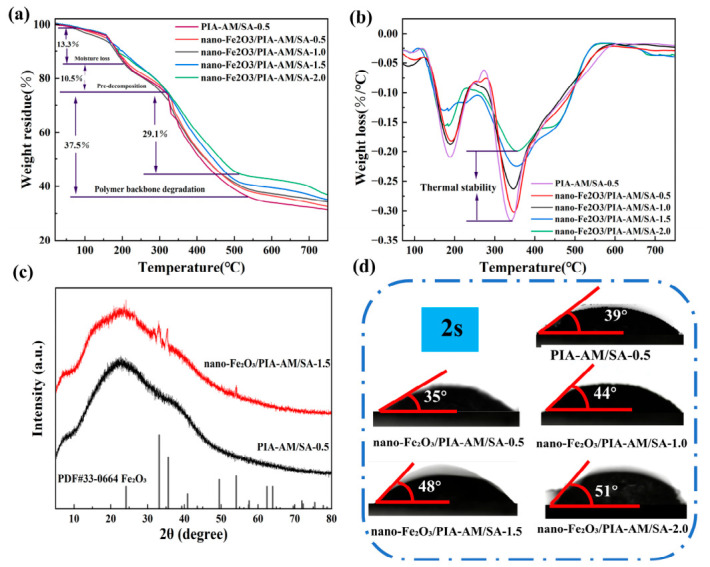
TG (**a**), DTG curves (**b**), XRD (**c**) and water contact angle images (**d**) of PIA-AM/SA and nano-Fe_2_O_3_/PIA-AM/SA composites.

TGA and DTG curves of the hydrogels are displayed in Figure 3a,b. Three distinct weight-loss stages were observed for all samples. The first stage covers within the temperature range from room temperature to 200 °C, where a weight loss of 13.3% was assigned to the evaporation of free and bound water. The second stage spans from 200 °C to 300 °C, during which a weight loss of 10.5% was attributed to the pre-decomposition of small molecular segments and functional side groups. The third stage, extending from 300 °C to 700 °C, represents the primary decomposition phase of the polymer backbones. A weight loss of 37.5% was observed for PIA-AM/SA-0.5, whereas only 29.1% was recorded for nano-Fe_2_O_3_/PIA-AM/SA-2.0. The DTG curves confirmed that the main decomposition peak of PIA-AM/SA-0.5 occurred at the lowest temperature with maximum intensity. As the IA content increased, the peak gradually shifted to higher temperatures, accompanied by a decrease in intensity. The enhanced thermal stability was ascribed to the elevated crosslinking density induced by higher IA content, which formed a stable coordination structure with fixed nano-Fe_2_O_3_ and mitigated the thermal degradation of polymer chains.

XRD patterns of the hydrogels were presented in Figure 3c. The blank hydrogel PIA-AM-0.5 exhibits a broad amorphous pattern devoid of distinct diffraction peaks. Characteristic diffraction peaks of Fe_2_O_3_ appeared at 2θ values of 33.2°, 35.6° and 54.1° in the composite hydrogel. The diffraction peaks of nano-Fe_2_O_3_/PIA-AM/SA-1.5 were well matched with the standard JCPDS card (PDF#33-0664) of α-Fe_2_O_3_, confirming the successful incorporation of crystalline Fe_2_O_3_ into the hydrogel matrix. The crystal structure of nano-Fe_2_O_3_ remains well preserved throughout the polymerization process. Additionally, the amorphous structure of the organic polymer network was maintained without obvious disturbance.

Contact angle measurements assess the surface wettability of the hydrogels, as illustrated in Figure 3d. The contact angle of the blank gel PIA-AM-0.5 was recorded at 39°. An increase in IA content correspondingly elevated the contact angle. All hydrogel products maintained contact angles below 90°, demonstrating favorable hydrophilicity. The contact angle increased from 35° to 51° with increasing IA content, indicating a gradual decrease in hydrophilicity. This trend was consistent with the swelling behavior: samples with smaller contact angles exhibited higher equilibrium swelling ratios, confirming the positive correlation between hydrophilicity and water absorption capacity. This excellent hydrophilicity established a robust foundation for the superior water absorption and swelling properties of the gels in soil environments.

### 2.2. Evaluation of pHpzc

**Figure 4:**
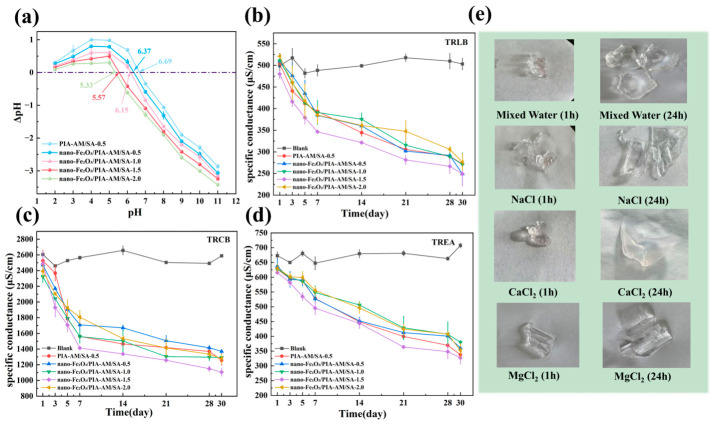
(**a**) pHpzc of hydrogels, electrical conductivity in soils of (**b**) TRLB, (**c**) TRCB, (**d**) TREA, and (**e**) displays photos of different hydrogels after swelling in various solutions.

In a low ionic strength aqueous environment, the hydrogel’s surface charge characteristics varied with pH, which could be controlled by adjusting the pH level [23]. Figure 4a displays the pHpzc results for a series of hydrogels. The pHpzc serves as a key indicator of the surface charge balance of particles. An increase in the content of COOH groups within the hydrogel leads to greater dissociation and the generation of COO^−^, resulting in a higher surface negative charge density, enhanced interparticle repulsion, and a more negative Zeta potential [24]. In this study, nano-Fe_2_O_3_/PIA-AM/SA-2 exhibited the lowest pHpzc (5.33), indicating optimal colloidal dispersion stability, which aligned with the SEM observations of good particle dispersibility and the absence of agglomeration. Saline–alkali soil is typically alkaline (with pH values mostly exceeding 7), and its soluble salts are primarily composed of cations such as Na^+^ (a typical high-content cation), Ca^2+^, and Mg^2+^, along with anions including Cl^−^ and SO_4_^2−^. Given that the alkaline pH significantly exceeds the pHpzc of nano-Fe_2_O_3_/PIA-AM/SA-2, the hydrogel surface presents a high negative charge density and preferentially adsorbs cations via electrostatic interactions, primarily absorbing Na^+^ while also being capable of adsorbing Ca^2+^, Mg^2+^, and other cations.

### 2.3. Swelling Behavior and Water Retention Performance

**Figure 5:**
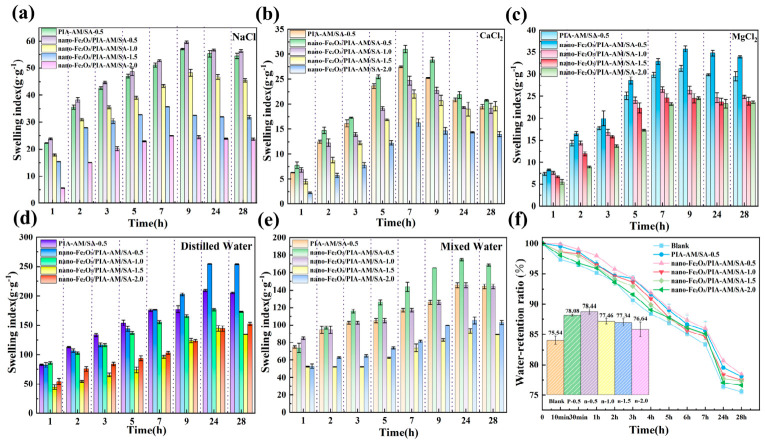
(**a**–**e**) represent the swelling images of hydrogels in solution of NaCl, CaCl_2_, MgCl_2_, Distilled Water, and Mixed Water, respectively, and (**f**) denotes the water retention image of hydrogels.

IA-based hydrogels represent promising superabsorbent materials. Figure 4e displays the swelling morphologies of the hydrogels in various aqueous solutions. At room temperature, the nano-Fe_2_O_3_/PIA-AM/SA hydrogels unveiled the fastest swelling rate within 7 h, achieving swelling equilibrium within 9 h and an equilibrium swelling ratio (ESR) exceeding 140 g·g^−1^ in deionized water (Figure 5). The variation in ESR correlated with the dosage of IA. As the IA content increased, the ESR initially rose gradually. However, excessive IA resulted in a more compact crosslinked network, which reduced the ESR. The nano-Fe_2_O_3_/PIA-AM/SA-0.5 exhibited the maximum ESR, ascribable to its optimal crosslinking degree and highest porosity.

The ESR was significantly affected by the ionic environment. It disclosed an ESR of 254.3 g·g^−1^ in deionized water, 174.63 g·g^−1^ in tap water, 59.66 g·g^−1^ in NaCl, 30.99 g·g^−1^ in CaCl_2_, and 35.72 g·g^−1^ in MgCl_2_ solution (Figure 5). The hydrophilic groups in the hydrogel network experienced electrostatic repulsion, which facilitated the extension of the hydrogel network and thus enhanced the water absorption capacity. The highest ESR appeared in ion-free water. The presence of metal cations in the solutions reduced the electrostatic repulsion among the hydrophilic groups, resulting in a significant decrease in ESR. Owing to their higher charge density, Ca^2+^ and Mg^2+^ displayed the strongest shielding effect on electrostatic repulsion, leading to the lowest swelling capacity.

Water retention capacity of the hydrogel was a core factor restricting soil productivity. It reflected that nano-Fe_2_O_3_/PIA-AM/SA-0.5 achieved a water retention index of 78.44% after 28 h (Figure 5f), which was significantly superior to other samples and the blank control group (75.54%). Its excellent water retention performance originated from the synergistic effect of well-developed pores and Fe^3+^-enhanced interfacial interactions. The optimal pore size enabled efficient free water storage, while Fe^3+^ polarization strengthened hydroxyl hydrogen bonding, improving bound water retention. By contrast, other samples showed structural drawbacks: nano-Fe_2_O_3_/PIA-AM/SA-1.5/1.0 had smaller pores limiting water storage; nano-Fe_2_O_3_/PIA-AM/SA-2 suffered from agglomeration and blocked pores due to excessive IA. The Fe-free PIA-AM/SA-0.5 exhibited weak hydrogen bonding. Overall, nano-Fe_2_O_3_/PIA-AM/SA-0.5 balanced water storage and retention, showing the best performance.

### 2.4. Modulatory Effects on pH and Electrical Conductivity in Soils

**Figure 6:**
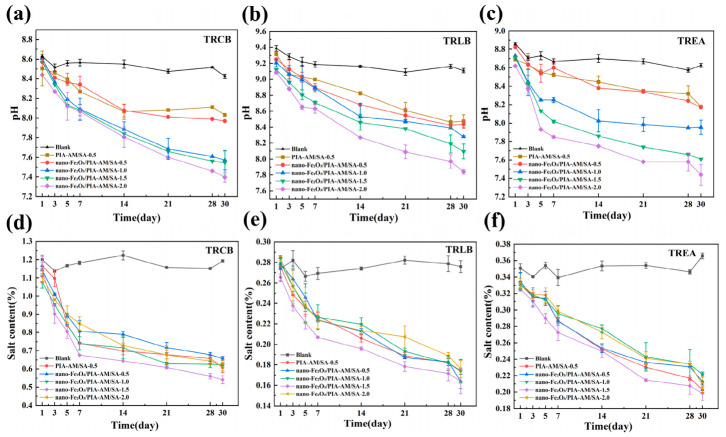
Effects of hydrogel on change in pH and salt content in TRCB (**a**,**d**), TRLB (**b**,**e**) and TREA (**c**,**f**) soil.

As depicted in Figure 6a–c, soil pH varied across different regions. The pH decreased from 8.44 to 7.40 in TRCB soil, dropped from 9.09 to 7.84 in TRLB soil, and declined from 8.60 to 7.44 in TREA soil. Nano-Fe_2_O_3_/PIA-AM/SA-2 unveiled the optimal pH regulation efficiency among all soil samples. This superior performance arose from the synergistic effect of high carboxyl density and Fe^3+^-mediated activation. Nano-Fe_2_O_3_/PIA-AM/SA-2 contained the highest IA content, which maximized surface carboxyl groups. These groups acted as key active sites, releasing H^+^ to neutralize soil OH^−^. Compared with lower IA formulations, it provided more reactive carboxyl groups for effective alkalinity neutralization. Additionally, Fe^3+^ weakened the H^+^ dissociation barrier, accelerating the neutralization reaction. With these combined advantages, it effectively reduced soil pH, adjusting highly alkaline TRLB soil and normalizing pH in TRCB and TREA within 30 days. This highlights its potential for saline–alkali soil improvement.

All hydrogel products manifested a significant reduction in electrical conductivity (EC) across three types of soils with varying salinization levels (Figure 4b–d). Among these, nano-Fe_2_O_3_/PIA-AM/SA-1.5 demonstrated the most pronounced regulatory performance (*p* < 0.05). For instance, the TRLB soil exhibited an EC reduction from 480 μS/cm to 249.27 μS/cm, representing a decrease of 48.07%. In TRCB soil, the EC decreased by 46.87%, while TREA soil experienced an EC reduction rate of approximately 55.47%. These consistent reductions across different salinized soils highlight the broad applicability of the treatments.

These results indicated that the regulatory effects of nano-Fe_2_O_3_/PIA-AM/SA-2.0, nano-Fe_2_O_3_/PIA-AM/SA-1.0, nano-Fe_2_O_3_/PIA-AM/SA-0.5, and PIA-AM/SA-0.5 on soil EC were inferior to that of nano-Fe_2_O_3_/PIA-AM/SA-1.5. This inferior performance was primarily attributed to the synergistic imbalance between composition and performance. Specifically, nano-Fe_2_O_3_/PIA-AM/SA-2 agglomerated due to excessive IA, resulting in encapsulated active sites. In contrast, nano-Fe_2_O_3_/PIA-AM/SA-1.0 and nano-Fe_2_O_3_/PIA-AM/SA-0.5 possessed insufficient functional active sites owing to inadequate IA. PIA-AM/SA-0.5 disclosed poor structural stability and weak charge interactions, which result from a lack of Fe, further diminishing its salt control efficiency. The positive correlation between EC and electrolyte ion concentration in the soil liquid phase suggested that higher ion concentrations led to stronger electrical conductivity. The effective salt control of nano-Fe_2_O_3_/PIA-AM/SA-1.5 derived from the synergistic effects of structure–interface interactions. Its tailored ratio of Fe to aluminosilicate created high-density active sites dominated by hydroxyl (-OH) and carboxyl (-COOH) groups. Furthermore, its mesoporous structure aligned with the diffusion radius of soil ions such as Na^+^ and Cl^−^, facilitating the efficient capture of conductive ions through physical adsorption and chemical complexation, thereby reducing the total ion concentration in the solution. The regulated surface charge density and potential characteristics facilitated the formation of electrostatic attraction within the electrical double layer with soil ions. Additionally, free salt ions could be fixed in mesopores or converted into solid-bound forms in the soil through a charge-balanced ion exchange mechanism, which accelerated ion migration and fixation. Notably, nano-Fe_2_O_3_/PIA-AM/SA-1.5 exhibited excellent adaptability across three soils with varying degrees of salinization. Despite differences in initial salt content, texture, and pore structure, it effectively removed ions through the synergistic regulation of structure and performance, exhibiting superior regulatory effects on electrical conductivity (EC).

### 2.5. Effects on Contents of Salt and Organic Matter in Soils

As shown in Figure 6d–f, the salt contents of the three soil samples decreased to 0.55, 0.16, and 0.19, respectively, with nano-Fe_2_O_3_/PIA-AM/SA-1.5 demonstrating the highest salt reduction efficiency. This advantage stems from the optimal IA dosage, which balances carboxyl density, crosslinking degree, and pore structure. It avoids insufficient adsorption sites at low IA and excessive crosslinking at high IA, creating a well-adapted pore structure for salt ion adsorption across different saline soils. It ultimately efficiently accomplished the selective adsorption of salt ions in all regions and achieved the optimal salt reduction performance. This outcome was consistent with the variation trend of EC, further verifying the stability of the salt reduction effect of nano-Fe_2_O_3_/PIA-AM/SA-1.5 on saline–alkali soil.

**Figure 7:**
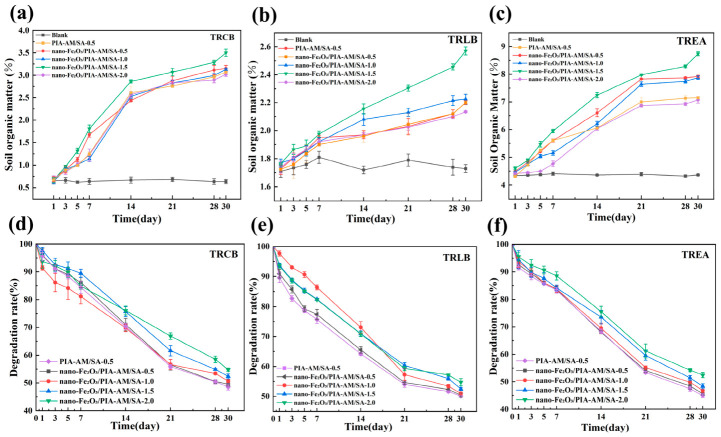
Effects of hydrogel on the content of organic matter and degradation rate in TRCB (**a**,**d**), TRLB (**b**,**e**) and TREA (**c**,**f**) soil.

The organic matter contents were increased from 0.67 to 3.5, 1.77 to 2.57, and 4.61 to 8.74 in TRCB, TRLB, TREA soil (Figure 7a–c), respectively. Nano-Fe_2_O_3_/PIA-AM/SA-1.5 exhibited the excellent effect on improving the organic matter content of soil samples. The moderate pore structure efficiently retains both endogenous and exogenous organic matter, such as plant residues, within the soil environment, thereby preventing the loss of organic matter that can occur in low-IA materials with excessively loose pores. However, the high-IA content of the high-IA hydrogel resulted in overly dense pores, which hindered the penetration of organic substances. This configuration provided adequate storage space for soil samples with relatively low initial organic matter content, specifically TRCB and TRLB. The surface carboxyl groups interact with the functional groups of organic matter in the soil samples through hydrogen bonding and electrostatic interactions. The optimized density of carboxyl groups not only prevented the desorption of organic matter but also mitigated structural densification that could arise from an excess of carboxyl groups, which would restrict contact. Furthermore, the slow and controllable degradation process preserved the organic matter immobilization structure within the soil samples and facilitated the release of small-molecule substances, such as organic acids, which nourish microorganisms. Microbial metabolism subsequently converted organic debris in the soil samples into immobilizable organic matter, leading to a significant enhancement in the organic matter content of barren soils.

### 2.6. Evaluation of Degradation Rate and Performance Comparison

After 30 days of degradation, the degradation rates (Figure 7d–f) of PIA-AM/SA-0.5 in soils of TRCB, TRLB, and TREA reached 50.1%, 48.56%, and 44.95%, respectively. Its degradation half-life was calculated to be approximately 30~32 days, while the half-lives of the nano-Fe_2_O_3_/PIA-AM/SA series ranged mostly from 45~60 days. This half-life can be precisely tailored to crop growth cycles by adjusting formulation parameters such as IA content.

For instance, PIA-AM/SA-0.5 is suitable for short-cycle leafy vegetables facilitating rapid degradation after harvest. In contrast, the half-life can be extended to 40 to 60 days for long-cycle crops, such as wheat and corn, by elevating the IA content. This hydrogel consists of biocompatible itaconic acid, natural sodium alginate and trace nano-Fe_2_O_3_, and ultimately decomposes into microbe-available organics and stable iron oxide. Itaconic acid substitution and nano-Fe_2_O_3_ coordination inhibit acrylamide release, while slow degradation prevents harmful accumulation. Acrylamide is further degraded into non-toxic small molecules that can be absorbed by plants. Improved wheat growth and increased soil organic matter confirm no ecotoxicity. PIA-AM/SA-0.5 degraded the fastest because the loose crosslinked network formed by its low IA content facilitated microbial penetration and the action of hydrolases. The small-molecule organic substances generated via decomposition nourish microorganisms, creating a positive feedback loop of “favorable environment promoting reproduction increased microorganisms accelerating degradation”. Conversely, nano-Fe_2_O_3_/PIA-AM/SA-2.0 demonstrated the slowest degradation performance, attributed to the dense structure formed by excessive crosslinking from high IA content, which hindered microbial penetration and enzyme contact. Notably, all hydrogel products degraded fastest in TREA soil, owing to the relatively high microbial biomass in this soil.

As shown in Table 1, the prepared nano-Fe_2_O_3_/PIA-AM/SA hydrogel exhibits good comprehensive performance relative to reported hydrogels for agricultural use. It achieves a relatively high salt content reduction efficiency of 54.1%, showing good performance in saline soil remediation. Its swelling capacity of 254.3 g·g^−1^ is comparable to acrylic acid-based hydrogels and higher than conventional iron-modified hydrogels. The degradation half-life of 30 days is moderate, falling between those of polysaccharide-based and acrylic acid-based hydrogels. Overall, it shows balanced performance advantages for agricultural applications.

### 2.7. Evaluation of Hydrogel Application Efficiency and Underlying Action Mechanisms

**Figure 8:**
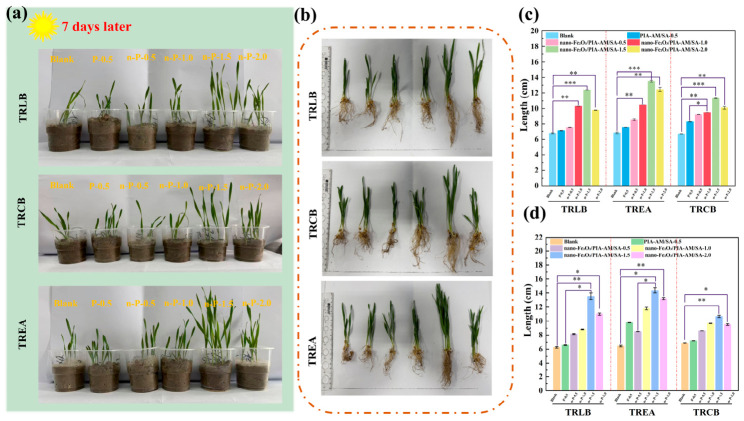
(**a**) The growth status, (**b**) the measurement diagram of rhizomes, (**c**) root length, and (**d**) stem length of wheat grown in three soils for seven days, * *p* < 0.05, ** *p* < 0.01, *** *p* < 0.001, denoting statistical divergence across all experimental cohorts.

The application of hydrogels in agriculture was investigated via a wheat planting experiment. This study employed a controlled experimental design comprising a blank control group, a positive control group treated with PIA-AM/SA hydrogel (without nano-Fe_2_O_3_), and multiple treatment groups exposed to nano-Fe_2_O_3_/PIA-AM/SA hydrogels at varying concentrations. Wheat seedlings were cultivated for seven days in three distinct soil substrates (TRLB, TREA and TRCB) to systematically evaluate the influences of nano-Fe_2_O_3_/PIA-AM/SA hydrogels on the growth-promoting efficacy. After 7 days, there were significant differences in the growth phenotypes of wheat seedlings among different treatment groups across the three environments. As observed in Figure 8a, the blank group displayed thin stems and sparse leaves. In contrast, the groups treated with nano-Fe_2_O_3_/PIA-AM/SA hydrogels (especially the nano-Fe_2_O_3_/PIA-AM/SA-1.5 and nano-Fe_2_O_3_/PIA-AM/SA-2.0 groups) exhibited stout stems, lush green leaves, and prominent growth advantages. Across the TRCB, TRLB, and TREA environments, the hydrogel-treated groups all proved to have growth performance than the blank group and PIA-AM/SA-0.5 group. These results demonstrated that nano-Fe_2_O_3_/PIA-AM/SA hydrogels can stably promote the growth of wheat seedlings in diverse environments.

Figure 8a shows the 7-day growth status of wheat seedlings under different treatments, Figure 8b presents the measurement schematic for seedling roots and stems, and Figure 8c,d display the quantified root and stem length results across all experimental groups. Specifically, the average stem lengths of seedlings in the TRCB, TRLB, and TREA were 10.64 cm, 13.52 cm, and 14.38 cm, respectively, with corresponding average root lengths of 11.32 cm, 12.39 cm and 13.53 cm. The nano-Fe_2_O_3_/PIA-AM/SA-1.5 group unveiled significantly better seedling growth than other groups.

To clarify the statistical reliability of inter-group growth differences, SPSS (26.0) was used for one-way analysis of variance (ANOVA). It revealed that inter-group differences in stem length reached an extremely significant level (*p* < 0.01), while differences in root length were statistically significant (*p* < 0.05). Significant differences were observed in both stem and root lengths among the treatment groups (*p* < 0.05). This proved that its growth-promoting effect was not random but rather supported by stable statistical evidence. From the perspective of soil microenvironment regulation, insufficient water typically prompts seedlings to extend their roots in search of moisture while inhibiting stem elongation. But the hydrogel network of nano-Fe_2_O_3_/PIA-AM/SA-1.5 can retain soil moisture over an extended period and release nutrients gradually, thereby avoiding ineffective root consumption under drought stress and providing a continuous material basis for stem elongation. Notably, nano-Fe_2_O_3_/PIA-AM/SA-1.5 exhibited stable growth-promoting effects in soils from different regions, supported by statistical results. It can therefore be concluded that these hydrogel materials created a favorable habitat for seedlings by optimizing the soil water–fertilizer microenvironment, with their growth-promoting effects exhibiting both statistical robustness and practical stability in field applications.

**Figure 9:**
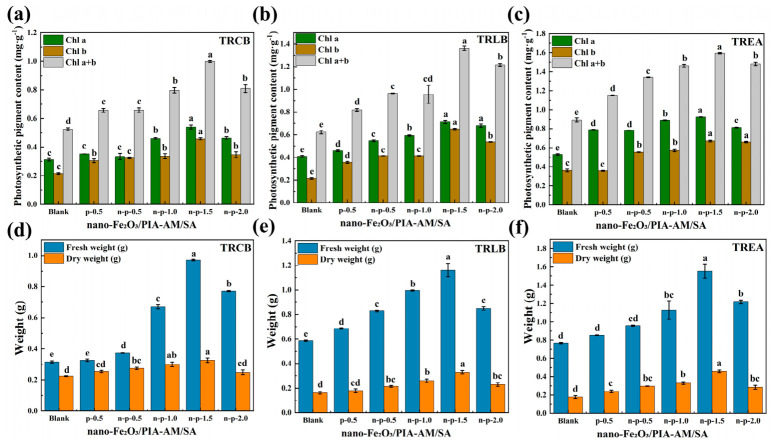
Effects of nano−Fe_2_O_3_/PIA−AM/SA hydrogel on wheat photosynthetic pigments and biomass in TRCB (**a**,**d**), TRLB (**b**,**e**) and TREA (**c**,**f**) soils. Different lowercase letters denote significant differences at *p* < 0.05.

Figure 9 demonstrates that all hydrogel treatments significantly increased wheat chlorophyll (Chl a, Chl b, Chl a + b) and biomass (fresh/dry weight) compared to the blank control in three saline–alkali soils, with all indicators showing a consistent trend of first increasing then decreasing with hydrogel concentration. The nano-Fe_2_O_3_/PIA-AM/SA-1.5 treatment achieved the optimal effect, outperforming the control and other treatments, while nano-Fe_2_O_3_-doped hydrogels were far more effective than the pure hydrogel (PIA-AM/SA-0.5). This improvement stems from the hydrogel’s strong water and nutrient retention, which mitigates saline–alkali stress-induced damage to the photosynthetic system; these findings confirm that nano-Fe_2_O_3_ doping effectively enhances the hydrogel’s stress-alleviating and growth-promoting capabilities.

### 2.8. Functional Mechanism

**Figure 10:**
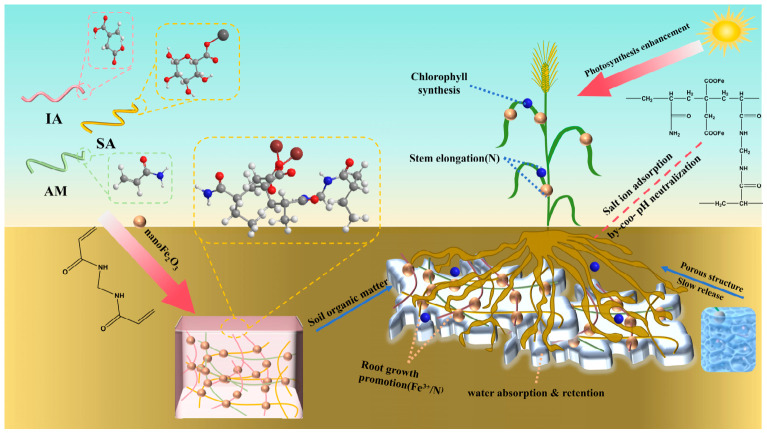
Schematic diagram of nano−Fe_2_O_3_/PIA−AM/SA composite hydrogel structure and action mechanism.

The nano-Fe_2_O_3_/PIA-AM/SA composite hydrogels facilitated saline–alkali soil remediation and wheat growth enhancement through a continuous porous three-dimensional crosslinked network created by a free-radical polymerization of IA, AM and SA. Nano-Fe_2_O_3_ was evenly distributed on the network skeleton, as illustrated in Figure 10. The molecular chains of the hydrogels contain essential active hydrophilic groups like carboxyl and hydroxyl, which neutralize excess soil OH^−^ by dissociating H^+^, regulating pH mildly, and capturing free Na^+^, Ca^2+^, and Mg^2+^ through electrostatic adsorption and coordination.

This process reduced soil electrical conductivity and total salt content, thereby alleviating crop osmotic stress. The hydrogel sporous configuration allowed for effective water absorption and retention, creating microspaces for soil nutrients and microorganism adhesion and storage to enhance soil microecology. The inclusion of loaded nano-Fe_2_O_3_ provides a micronutrient effect by supplying iron to wheat, facilitating the absorption and transformation of N, P, and other mineral nutrients by roots to overcome hindered nutrient uptake in saline–alkali soil. Furthermore, the hydrogels exhibited controllable degradability, gradually releasing small-molecule organics like organic acids and polysaccharides to boost soil organic matter content continuously, nourish soil microorganisms, and optimized the rhizosphere microenvironment. The combined effects of structural stabilization, saline–alkali regulation, water and fertilizer retention, nutrient provision, and microecological optimization led to a comprehensive enhancement of physicochemical properties in saline–alkali soil. This, in turn, significantly promoted the growth of roots and stems in wheat seedlings.

## 3. Conclusions

In summary, to synergistically enhance water–hydrogel–fertilizer efficiency and improve soil adaptability, this work proposed formula-controllable functional hydrogels regulation strategies. Degradable and environmentally friendly IA-based nano-iron composite hydrogels (nano-Fe_2_O_3_/PIA-AM/SA series) were designed and synthesized. The crosslinked network of the hydrogels was constructed by regulating the dosage of IA. The results reflected that the crosslinked structure of the hydrogel could transition from a loose configuration to a dense configuration, thereby fulfilling the performance requirements of different crop growth cycles. The hydrogels exhibited excellent swelling properties (equilibrium swelling degree of 140–254 g·g^−1^) and controllable degradability (half-life of 30–70 days), which endowed them with remarkable water retention and nutrient slow-release capabilities. These properties not only extended the irrigation interval in arid soils but also improved soil structure and nourished microorganisms, optimizing the rhizosphere environment. Furthermore, these hydrogels promoted crop growth and met the regulatory requirements of different crop growth cycles. Consequently, the IA-based composite hydrogels demonstrated multiple benefits, including water retention, soil improvement, and growth promotion, while remaining eco-friendly and residue-free after degradation. These novel agricultural functional materials show good potential for application in water-saving agriculture, soil remediation, and related fields. For further study, we will optimize large-scale synthesis processes, conduct long-term field trials, and explore compound applications with other soil amendments to verify environmental safety and crop adaptability. With low synthesis cost, this material holds promising application prospects in arid/semiarid saline–alkali lands, coastal saline soils, facility agriculture, and degraded mine land restoration.

## 4. Materials and Methods

### 4.1. Materials

All reagents employed in this study were of analytical grades. Nano-Fe_2_O_3_ and IA were procured from Dibo Biotechnology Co., Ltd. (Shanghai, China) and Chuangying Chemical Co., Ltd. (Jinan, China), respectively. AM and potassium persulfate were acquired from Zhiyuan Chemical Reagent Co., Ltd. (Tianjin, China). N, N′-methylene bisacrylamide was obtained from Bide Pharmaceutical Technology Co., Ltd. (Shanghai, China). Moreover, sodium hydroxide was sourced from Yatai United Chemical Co., Ltd. (Wuxi, China), and SA was purchased from Mingyue Seaweed Group Co., Ltd. (Qingdao, China).

### 4.2. Synthesis of Hydrogel Materials

**Figure 11:**
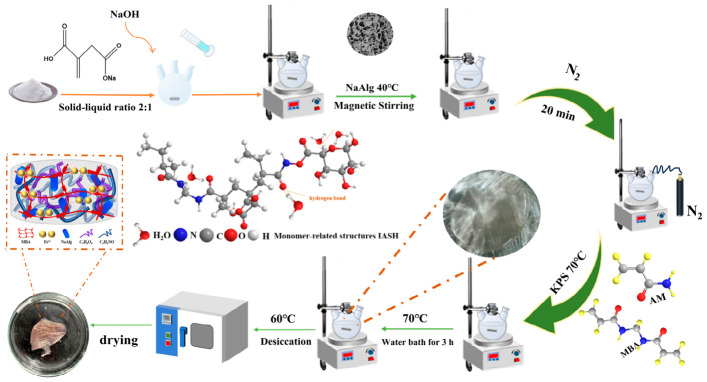
Flowchart of product synthesis.

Hydrogel materials were synthesized through the free-radical polymerization of itaconic acid (IA), acrylamide, and sodium alginate. Initially, 0.5 g of IA and 0.25 g of sodium hydroxide were accurately weighed and dissolved in 20 mL of distilled water. Subsequently, 10 mL of a nano-Fe_2_O_3_ solution (0.3 mmol·L^−1^) and sodium alginate (0.08 mol·L^−1^) were added successively while stirring at 70 °C. A nitrogen line was connected, adjusted to a flow rate of 0.5 L·min^−1^, and bubbled for approximately 15 min to prevent oxygen from inhibiting free-radical polymerization. Next, acrylamide (1.40 mol·L^−1^), N, N′-methylene bisacrylamide (0.01 g), and potassium persulfate (0.02 g) were sequentially added to the flask. The solution was then stirred for 3 h until a highly viscous elastic gel product was obtained. Finally, the hydrogel composite was oven-dried at 60 °C, yielding the hydrogel product of nano-Fe_2_O_3_-loaded poly (itaconic acid-co-acrylamide)/sodium alginate (nano-Fe_2_O_3_/PIA-AM/SA). The resulting hydrogel products, varying in IA content (0.5 g, 1.0 g, 1.5 g, and 2.0 g) were labeled as nano-Fe_2_O_3_/PIA-AM/SA-0.5, nano-Fe_2_O_3_/PIA-AM/SA-1, nano-Fe_2_O_3_/PIA-AM/SA-1.5, and nano-Fe_2_O_3_/PIA-AM/SA-2, respectively. Nano-Fe_2_O_3_ was utilized to functionally modify the hydrogel. The product without nano-Fe_2_O_3_ served as a comparison and was designated as PIA-AM/SA-0.5. The synthetic flowchart was illustrated in Figure 11.

### 4.3. Determination of pHpzc

The point of zero charge (pHpzc), an essential characteristic of the hydrogel, denotes the pH at which the positive and negative charges on the surface of a solution achieve equilibrium [31]. A total of 50 mL of 0.01 mol·L^−1^ NaCl solution was transferred to an Erlenmeyer flask, and the initial pH of each solution (designated as pH_0_) was adjusted to values ranging from 2.0 to 11.0 using 0.1 mol·L^−1^ HCl or NaOH solution. Subsequently, 0.05 g of the hydrogel was weighed and added to the flask, with the resulting pH recorded as pH_1_. The flasks were then placed in an air-bath shaker at 25 °C and agitated at 140 rpm for 48 h. The equilibrium pH of the solution was noted as pH_2_ [32]. A curve was generated with the initial pH_0_ plotted on the x-axis and ΔpH (pH_2_ − pH_1_) on the y-axis. The pH corresponding to the intersection of this curve with the line y = 0 was designated as the hydrogel’s pHpzc.

### 4.4. Measurement of Swelling Index and Water Retention Capacity

The swelling index (g·g^−1^) was performed following the literature with modifications [33]. Hydrogel materials (0.1 g) were cut into small pieces and immersed individually in deionized water, tap water, 0.9% NaCl, 0.9% CaCl_2_, and 0.9% MgCl_2_ solution at room temperature until reaching a constant weight. The hydrogel samples were periodically removed during soaking (at 1, 3, 5, 7, 9, 24, 28 h) and weighed. The swelling index was calculated for the hydrogel products using Equation (1). Subsequently, the saturated hydrogel products were removed for natural drying at specified intervals and weighed to determine the water retention index with Equation (2).

(1) $$Swelling\;index=\frac{m_{1}}{m_{2}}\times 100\%$$

(2) $$Water\;retention\;index=\frac{m_{a}}{m_{b}}\times 100\%$$

where *m*_1_ and *m*_2_ represent the weight of the dry product and the water-swollen product, respectively. *m*_a_ (g) and *m_b_* (g) denote the weight of the hydrogel material remaining after natural drying and after saturation, respectively.

### 4.5. Measurement of pH, Electrical Conductivity, Contents of Salt and Organic Matter, and Degradation Rate in Soils

Three typical sandy loam soils (labeled as TRCB, TRLB and TREA) were collected from different locations at a depth between 0 and 30 cm from Alar in Xinjiang, China. TRCB (pH 8.44, highest salt content, 0.67% organic matter) simulates severely saline farmland; TRLB (pH 9.09, highest alkalinity, 1.77% organic matter) represents strongly alkaline farmland; and TREA (pH 8.60, moderate saline–alkali properties, 4.61% organic matter) corresponds to the most common moderately saline–alkali farmland in the region. This gradient design allows a comprehensive evaluation of the hydrogel’s performance under varying saline and alkaline stresses. The soil samples were oven-dried at 105 °C for 24 h and subsequently passed through a 120-mesh sieve to standardize particle size distribution prior to further analysis. The pH, electrical conductivity and salt content were assessed on Days 1, 3, 5, 7, 14, 28, and 30 during the incubation period (n = 3). A total of 30 g of the three selected soils and 0.2 g of the hydrogel product were accurately weighed and homogenized thoroughly. Afterwards, the mixed soil (1 g) was taken out, distilled water was added at a soil-to-water ratio of 1:5 (*m*/*v*) and agitated in a shaker under 180 rpm for 30 min of oscillation. Subsequently, the pH, electrical conductivity and salt content were measured using a pH meter (Model FE28, Mettler Toledo, Greifensee, Switzerland) and an electrical conductivity meter (Model DDS-11A, Shanghai Leici Instrument Co., Ltd., Shanghai, China) [34,35], respectively. Moreover, the contents of organic matter were measured using potassium dichromate oxidation method [36].

The degradation rate was determined as follows. Hydrogel samples (2 g of dry weight) were wrapped in spandex filter screens and buried 3 cm below the surface of natural soils. The hydrogel samples were retrieved and weighed at intervals (Days 1, 3, 5, 7, 14, 21, 28, and 30). All experiments were performed in triplicate, and the degradation rate was calculated according to Equation (3).

(3) $$Degradation\;rate=\frac{m_{0}-m_{x}}{m_{0}}\times 100\%$$

where m_x_ represents the mass of the hydrogel at Day X, and *m*_0_ represents the initial mass of the hydrogel.

### 4.6. Hydrogel-Treated Wheat Seedling Cultivation Assay

In the crop planting experiment, the incubation conditions were set to the following: a constant temperature of 28 ± 2 °C, a relative humidity of 30 ± 3%, and a 12 h photoperiod [37]. Wheat seeds, uniform in weight and size, were selected for pretreatment. They were soaked in a 2% sodium hypochlorite solution for 5–10 min, rinsed three times with distilled water, and then incubated for 24 h. The three aforementioned soils served as the growth substrate. Prior to planting, the soils were fully moistened with distilled water and equilibrated for 24 h. Subsequently, the seeds were sown in containers (11 × 10 cm, 5 seeds per pot), and 0.2 g of the corresponding hydrogel product was added to each soil group and thoroughly mixed. The seeds were evenly spaced in the soil with a sowing depth of 1 cm, and approximately 10 mL of distilled water was applied every 2 days during the experiment. The crops were harvested at the 7-day seedling stage (n = 3), rinsed thoroughly with distilled water post-harvest, and gently blotted dry. The fresh weight of whole seedlings was weighed immediately. Chlorophyll a, chlorophyll b and total chlorophyll contents in fresh leaves were determined using the ethanol extraction method. Seedlings were then separated into roots, stems, and leaves, oven-dried at 70 °C to constant weight, and the dry weight was recorded, separated into roots, stems, and leaves, and then subjected to subsequent analysis [38].

### 4.7. Hydrogel Material Characterization

The PIA-AM/SA and nano-Fe_2_O_3_/PIA-AM/SA samples underwent comprehensive characterization using Fourier transform infrared spectroscopy (FTIR), Raman spectroscopy, X-ray diffraction (XRD), X-ray photoelectron spectroscopy (XPS), scanning electron microscopy with energy-dispersive X-ray spectroscopy (SEM-EDS), contact angle (CA) and thermogravimetric analysis (TGA) to examine their chemical structure, crystallinity, microstructure, elemental distribution, hydrophilicity, and thermal stability [39]. The changes in hydrogel crystallinity were analyzed using a D8 Advance diffractometer (Bruker Corporation, Karlsruhe, Germany) with Cu Kα radiation (λ = 0.154 nm, 2θ = 5–80°). Morphology and pore structure were examined with a JSM-6700F scanning electron microscope (SEM, JEOL, Tokyo, Japan), and the elemental distribution (Fe, C, O, Na) was qualitatively and quantitatively determined using the attached X-MaxN energy-dispersive X-ray spectroscopy (EDS) detector to confirm uniform nano-iron loading [40]. Characteristic peaks were detected and intercomponent interactions were verified via FTIR (Thermo Fisher Scientific, Waltham, MA, USA). Thermal stability and carbonization behavior were assessed via TGA (STA 449 F5, Netzsch GmbH & Co. KG, Selb, Germany) from 30 °C to 700 °C. The chemical states of surface elements were analyzed using XPS on an ESCALAB spectrometer (Thermo Fisher Scientific, USA) [41]. Insights into structural modifications and functional enhancement were collectively offered by these techniques, and a correlation between microstructure and improved soil conditioning performance was established [42]. These techniques collectively provided insights into structural modifications and functional enhancements, establishing a correlation between microstructure and improved soil conditioning performance.

### 4.8. Data Analysis

All experimental data were presented as mean ± standard deviation (SD), with error bars in the figures representing the standard deviation of the datasets. All experiments were conducted with three independent replicates (n = 3). One-way analysis of variance (ANOVA) followed by Duncan’s multiple range test was used to determine the statistical differences among groups at a significance level of *p* < 0.05. Significant differences between groups were indicated by asterisk markings.

## Figures and Tables

**Table 1:** Performance comparison of various hydrogels for saline-alkali soil remediation.

Hydrogel Type	Swelling Performance (g·g^−1^)	Salt Content Reduction (%)	Degradation Half-Life (Day)	References
nano-Fe_2_O_3_/PIA-AM/SA	254.3	54.16	30	Current work
Acrylic acid-based hydrogels	170.6	31.7	45	[25]
	272.3	42.24	33	[26]
Polysaccharide-based hydrogels	230.4	24.56	18	[27]
	212.8	49.34	22	[28]
Conventional iron-modified hydrogels	185.28	35.6	28	[29]
	167.43	32.1	50	[30]

## Data Availability

The original contributions presented in this study are included in the article. Further inquiries can be directed to the corresponding author.
